# Determination of the Risk Factors Contributing to the Development of Neuropsychiatric Lupus in a Systemic Lupus Erythematosus Cohort

**DOI:** 10.7759/cureus.20129

**Published:** 2021-12-03

**Authors:** Adegbenga A Bankole, Taskeen R Kazmi, Alyssa R Strazanac

**Affiliations:** 1 Rheumatology, Virginia Tech Carilion School of Medicine (VTCSOM), Roanoke, USA; 2 Rheumatology, Carilion Clinic, Roanoke, USA; 3 Medicine/Rheumatology, Wake Forest School of Medicine, Winston-Salem, USA

**Keywords:** systemic lupus erythematosus with polyneuropathy, neurology and psychiatric disorders, neurology, psychiatry, neuropsychiatric lupus, complements, antiphospholipid antibody, systemic lupus erythematosus

## Abstract

Background

Systemic lupus erythematosus (SLE) is a chronic autoimmune disease with a complex, varied clinical presentation that is both more common and has poor outcomes in women of color. SLE outcomes also seem to be influenced by socioeconomic factors. Neuropsychiatric lupus (NPL) is a common manifestation of SLE that is difficult to diagnose and treat and has poor clinical outcomes. There is no clear relationship between NPL and SLE-related autoantibodies, and this contributes to the difficulty in diagnosing NPL. As a result, NPL is a significant contributor to morbidity and mortality in patients with SLE.

Objective

The purpose of this study was to examine the relationship between serological and socioeconomic factors in the development of NPL in our patient cohort and determine the risk factors for the development of NPL.

Methods

This was an SLE single-center, retrospective chart review study that was performed at a university-based tertiary referral center. Patients aged 18 and older who meet the American College of Rheumatology (ACR) 1997 criteria and were seen between June 1st, 2015, and June 1st, 2019, were included in this study. Overall, 629 patients with SLE were identified, and 263 patients were included. Demographic and serological data were collected. Supplemental socioeconomic information for each zip code in Southwest Virginia was obtained from the United States Government Census website. Continuous variables were analyzed using the T-test or Mann-Whitney U test. Categorical variables were analyzed using chi-square tests or Fisher’s exact tests. Statistical analysis was performed using SAS9.4, and p-value < 0.05 was considered statistically significant.

Results

We reviewed a number of risk factors including age, sex, race, and median household income (MHI), noting no statistical relationship between these factors and the diagnosis of NPL. We did find that the presence of antiphospholipid antibodies (aPL) was significantly associated with a diagnosis of NPL and that complement 4 (C4) levels trended toward statistical significance.

Conclusion

In our cohort of patients, there was no relationship between age, sex, race, and median household income, and the diagnosis of NPL. There was a statistically significant relationship between aPL and the diagnosis of NPL. Other SLE-related antibodies showed no statistical relationship with the diagnosis of NPL. Although not statistically significant, there was a trend toward significance between complement 4 (C4) levels and the diagnosis of NPL.

## Introduction

Systemic lupus erythematosus (SLE) is a chronic, inflammatory, systemic autoimmune disease that often affects multiple different organs [1]. Although any organ can be involved, the skin and kidneys are more commonly affected. SLE can also present with symptoms related to the nervous system, including the autonomic, peripheral, and central nervous systems. The constellations of symptoms related to the nervous system form what is called neuropsychiatric lupus (NPSLE) and can be severe or even life-threatening. It has been estimated that up to 30% of all neuropsychiatric events occurring in patients can be attributed to SLE [2]. Clinically, NPSLE is one of the most complex and challenging features of SLE. Even in patients with well-established SLE, it is often difficult to ascribe with certainty the various array of neurological symptoms that can occur to NPSLE. To assist with the case definition of NPSLE, the American College of Rheumatology (ACR) has developed definitions and criteria for neurological symptoms that make up NPSLE [3]. These case definitions also provide the common NPSLE nomenclature used clinically and in research and allow for a common understanding of this disease.

Despite our current level of understanding of SLE, its pathogenesis, and the recent advancements in the treatment of SLE, we lack a full understanding of what autoantibodies are involved in the development of NPSLE. A disease activity tool that meets the needs of our patients and their care providers is also not available. The interpatient variability of symptoms, attribution difficulty of the symptoms to NPSLE, and lack of specific biomarkers for either the diagnosis of NPSLE or disease activity make NPSLE both difficult to diagnose and treat [4]. The lack of a specific biomarker may suggest that NPSLE is multifactorial, including both ischemic and neuro-inflammatory causes and not necessarily just related to autoantibodies [5]. This is not unusual as, although SLE is defined by the presence of autoantibodies, these autoantibodies are not always symptom- or organ-specific.

NPSLE does form part of the overall diagnostic criteria for SLE that was developed by the ACR in 1982, which was subsequently updated collaboratively with other stakeholders, first in 1997 and most recently in 2019 [6]. In this most recent diagnostic criteria, NPSLE is one of seven clinical domains that make up SLE [7].

Given the varied range of symptoms that NPSLE may present with, it can be a challenge to diagnose and perform appropriate diagnostic testing [8]. This is further complicated by the lack of biomarkers even when NPSLE is suspected [9]. The difficulty in appropriately ascribing the neuropsychiatric symptoms to NPSLE makes both diagnosis and treatment difficult and has led to poor patient outcomes we have observed in patients with NPSLE. This gap in care that our SLE patients experience should spur the rheumatology community to action to meet this need.

This article was previously presented as a meeting abstract at the 2021 Pan American League of Associations for Rheumatology (PANLAR) Annual Scientific Meeting on August 12th-15th, 2021.

## Materials and methods

This is a single-center, retrospective chart review study based in Southwest Virginia. Carilion Clinic/Virginia Tech Carilion School of Medicine (CC/VTCSOM) is a university-/academic-based tertiary referral center that provides care to the community of Southwest Virginia and has an active lupus clinic involved in various forms of research. The Carilion Clinic Institutional Review Board (IRB) approved that the risk of this study was minimal, and it was granted a full HIPAA waiver.

Our study population included any patient followed by the Rheumatology Department at VTCSOM with a diagnosis of SLE who were eligible for enrollment. The inclusion criteria are patients aged 18 and older who meet the ACR 1997 criteria. Patients actively followed by rheumatologists within the CC/VTCSOM system between June 1st, 2015, and June 1st, 2019, on immunosuppressive medications were eligible to be enrolled. To exclude confounders, patients with preexisting neurological, psychiatric, and mental health diseases (other than mood disorders such as depression and anxiety) that were not attributed subsequently to NPSLE were excluded.

The technology services group at CC/VTCSOM identified the electronic medical records (EMRs) for 629 patients with a diagnosis of SLE based on the International Classification of Diseases codes who were actively followed in the rheumatology clinic. Members of the research team examined the charts and confirmed 263 patients who met the ACR diagnostic criteria for SLE and the inclusion and exclusion criteria for this study. These 263 patients were included in our research database, and a more detailed chart examination was performed. We extracted relevant clinical and laboratory data from the time of diagnosis of SLE was made and at each further clinical encounter. The data extracted included autoantibodies, NPSLE symptoms (symptoms that make the ACR case definition of NPSLE, including seizures, psychosis, mononeuritis multiplex, myelitis, peripheral or cranial neuropathy, and cerebritis/acute confusional state), and immunosuppressive medications and doses, and patient demographic data, such as sex, age, race, and zip codes, were also collected.

As expected, the group was mostly composed of women of childbearing age, with a higher proportion of minorities and people of a lower socioeconomic status (SES). Supplemental data related to median household income (MHI), which reflected the SES of the patients, were obtained using ZIP code-related data from the United States Government Census website [10].

All laboratory tests performed at CC/VTCSOM were collected on-site and processed through our reference laboratory systems at Quest Diagnostics in a standardized method.

Data were subsequently analyzed by an assigned statistician. No patient with SLE who met our inclusion criteria and enrolled was excluded from the statistical analysis. Continuous variables were analyzed using the T-test or Mann-Whitney U test. Categorical variables were analyzed using chi-square tests or Fisher’s exact tests. Mental health variables were analyzed using McNemar’s tests. Statistical analysis was performed using SAS9.4, and p-value < 0.05 was considered statistically significant. There were no missing data items, and all patients in the cohort were included in the statistical analysis.

## Results

We identified a total of 263 patients with SLE who met the inclusion criteria, and their demographics are shown in Table 1. The majority of the patients were White (148, 54%), followed by African Americans (115, 43%), with only a small number of patients being Hispanics (6, 3%). This reflects the demographic breakdown of Southwest Virginia, an area of the USA with a higher proportion of African Americans (19%) as compared with the USA in general (12%) based on data from the US Census Bureau, Virginia Department of Health. As expected, our SLE cohort was composed of a higher proportion of African Americans as compared with that of the general population in the Southwest Virginia region.

We examined several factors in relation to the diagnosis of NPSLE. We found no statistical relationship between patient demographics including age, sex, race, and MHI, and the diagnosis of NPSLE in patients with SLE (Table 2). We further analyzed the relationship between MHI and the diagnosis of NPSLE across a range of median incomes and found no statistically significant relationship between these income brackets and NPSLE.

The analysis of SLE-specific autoantibodies (double-stranded DNA, Smith, ribonucleoproteins, and Sjogren’s syndrome-related antigen A and B) did not reveal a statistically significant association between these autoantibodies and NPSLE. There was a statistically significant association between the presence of antiphospholipid antibodies (aPL) and NPSLE (Table 3). Further analysis of aPL and patient demographics did not reveal a significant relationship between aPL positivity and race, sex, age, or MHI across the same ranges of incomes. We also examined the complement levels and found that, although not statistically significant, complement component 4 (C4) did show a trend toward significance. There was no statistically significant relationship between NPSLE and complement component 3 (C3).

We did find a statistically significant relationship between glucocorticoid use and the presence of NPSLE, but there was no relationship between hydroxychloroquine and immunosuppressive therapy and the presence of NPSLE in our SLE cohort.

**Table 1:** Patient Demographics ANA: antinuclear antibody Anti-Sm: anti-Smith antibody aPL: antiphospholipid antibody ds-DNA: anti-double-stranded DNA antibody C3: complement 3 C4: complement 4

Characteristics	All (N = 263)
Age (mean ± SD)	36.5 ± 13.5
Female	85.1% (222/261)
Race	
White	57.4% (148/258)
Black	36.0% (93/258)
Hispanic	2.3% (6/258)
Other	4.2% (11/258)
Median household income ($K, (mean ± SD))	44.3 ± 14.4
<26.2K	14.30%
26.2K–56.5K	62.90%
>56.5K	22.90%
Any antibody	93.3% (238/255)
ANA	90.3% (233/258)
Anti-ribosomal P	16% (38/238)
Anti-histone	36.7% (90/245)
Anti-Sm	27.6% (68/246)
aPL	15.8% (38/241)
ds-DNA	58.2% (145/249)
Low C4 (L/N)	42.6% (106/249)
Low C3 (N/L)	47% (118/251)

 

**Table 2:** Patient Characteristic and the Diagnosis of Neuropsychiatric Lupus No: SLE without NPL Yes: neurological symptoms ascribed to NPL

Characteristics	No (n = 166)	Yes (n = 82)	p-value
Age (mean ± SD)	36.6 ± 13.4	36.1 ± 13.5	0.83
Female	83.7%	86.4%	0.58
Race			0.22
Caucasian	54.9%	65.0%	
Black	37.2%	32.5%	
Hispanic	3.0%		
Other	4.9%	2.5%	
Median household income ($K, (mean ± SD))	43.6 ± 14.2	45.8 ± 14.8	0.27
	15.4%	12.0%	0.73
$26,200–$56,500	62.3%	62.7%	
>$56,500	22.2%	25.3%	

**Table 3:** Serology and Neuropsychiatric Lupus Relationship between antiphospholipid antibody and complement C4 and symptoms of neuropsychiatric lupus

Serology		Neuropsychiatric lupus		Total	Sensitivity	Specificity	p-value
		No	Yes				
Antiphospholipid antibody	No	145	53	198			
	Yes	15	23	38			
Total		160	76	236	30%	91%	<0.01
Complement component 4	No	100	38	193			
	Yes	64	39	103			
Total		164	77	241	51%	61%	0.09

## Discussion

SLE occurs more frequently and is more severe in younger women of color as compared to other groups [11]. NPSLE is a significant cause of morbidity in SLE and can be seen in up to 50% of all patients with SLE [12]. Despite the ACR’s case definitions of NPSLE and how frequently NPSLE may occur, it is still difficult to determine and confirm that the neuropsychiatric symptoms seen in these patients are actually related to SLE [8]. Given these attribution difficulties and to avoid potential confounders that would affect the interpretation of our data, we excluded all patients with primary neurological disorders. Patients with psychiatric and mental health diseases other than primary mood disorders (depression and anxiety) were also excluded. Mood disorders do occur commonly in the general population, with studies confirming a lifetime morbid risk and 12‐month prevalence estimates of generalized anxiety disorder in the USA of 9.0% and 2.0% [13], respectively. In addition, it does seem that mood disorders such as depression are becoming more frequent in people aged 12 and above in the USA and that these diseases are more common in young people and women [14] who also happen to be the most at risk for SLE. Excluding patients with mood disorders would have significantly reduced the total number of patients in this study. Despite the frequency of mood disorders in the general population, both depression and anxiety are also more common in people with SLE [15,16]. Mood disorders are also a common manifestation of NPSLE and are the second commonest manifestation of NPSLE [5]. There is also an increase in the diagnosis of mood disorder following the diagnosis of SLE, even in the absence of NPSLE. This temporal association between the increasing diagnosis of depression and anxiety, and SLE is well known and has been reported in a number of studies [17], although the exact cause of this association is not fully understood. We did also find an increase in the frequency of diagnosis of depression and anxiety in our cohort of patients. In our cohort, however, no cases of depression or anxiety were attributed to NPSLE.

The percentage of patients who develop NPSLE and their clinical presentation varies widely in the literature [18]. In general, the diagnosis and treatment of SLE and NPSLE can be a difficult and time-consuming task for the healthcare provider and a lifelong journey for the patient. The large variety of neurological symptoms, lack of confirmatory biomarkers [4,19], and small number of NPSLE treatment trials, most of which have varied and poor outcomes in responses to immunosuppressive therapy [20], make it difficult to identify and treat NPSLE in a manner that satisfies either the patient or the care provider. A number of studies have tried to identify reliable biomarkers, and if these can be identified, then confirmation of the diagnosis of NPSLE would become easier.

In our cohort, we found a significant relationship between the presence of aPL and NPSLE. Unterman et al. noted similar findings in a meta-analysis that was performed and confirmed an increased frequency of aPL in patients with NPSLE [21]. The current published data on aPL and NPSLE is not settled, with conflicting reports showing that aPL is both unrelated [22] and related [19] to NPSLE. Some of these conflicting reports may be related to the changes made to the case definition of NPSLE. Given the updated ACR case definition of NPSLE, it is harder to perform comparative analysis and studies that include cohorts from different eras.

As was noted in our study, a number of studies have confirmed that a low C4 is related to the diagnosis of NPSLE. However, we do know that C4 is related to SLE and that the activation of the complement cascade plays a central role in SLE, and monitoring of complement levels can be used to determine disease activity [23]. Therefore, we do not know if the low C4 observed is related to SLE activity or directly related to NPSLE. We do know now that monitoring of cell-bound complement activation products reflects more accurately SLE disease activity as compared to conventional serum C3 and C4 [24]. Some studies have also shown that complement levels in the cerebrospinal fluid (CSF) and the complement CSF/serum ratio may be a more accurate way of predicting and monitoring NPSLE [25]. More recent studies have suggested that a low C4 at the time of the diagnosis of SLE can be predictive of the development of severe NPSLE [26].

There appear to be no particular immunosuppressive medications that were preferred to treat NPSLE, as we found no relationship between immunosuppressive medications and NPSLE. This may reflect a lack of evidence as to where the more recently approved immunosuppressive medications for SLE fit in the treatment of NPSLE. Due to the heterogeneity of NPSLE, it is unlikely that one agent will meet all of our patient’s needs. A number of agents are currently being investigated, but the lack of drug development and subsequent approval by the regulators may reflect the heterogeneity in the causes and presentation of NPSLE [27], making one best drug for NPSLE difficult to develop. We did however find that there was higher use of prednisone in patients with NPSLE. This likely reflects treatment of active NPSLE rather than a causative relationship, although glucocorticoids may have neurological side effects that are similar in presentation to mood disorders and NPSLE [28]. Glucocorticoids are commonly used as a first-line treatment in SLE, with the dose and route of administration reflecting disease activity [29]. Rheumatologists often use moderate to high dose of glucocorticoids when SLE is active or affecting organs associated with high rates of morbidity or mortality [30].

Although it may not be applicable to other areas of the world, we did review the role of health insurance in NPSLE. As health insurance is mostly employer-based in the USA, we also examined MHI in relationship to NPSLE. Additionally, we also reviewed government-based insurance versus commercial health insurance, and in our cohort, we found no relationship between the type of health insurance and MHI in the diagnosis and treatment of NPSLE.

Although our cohort included a large number of patients with SLE, one of the weaknesses of our study was its retrospective nature. Although we were able to identify associations, we were unable to state the exact nature of the relationship.

## Conclusions

NPSLE can be a devastating condition for patients and is difficult for the care providers to both diagnose and treat. There are several possible pathogenic pathways that may lead to NPSLE, including ischemia, coagulopathy, antibody-related inflammation, complement activation, and deposition. Not all of the many symptoms of NPSLE respond to immunosuppressive therapy, sometimes calling doubt to the diagnosis that is sometimes made only clinically.

Further research is needed to determine exactly how aPL and C4 levels are associated with NPSLE, and what other biomarkers can be used to assist in the diagnosis of NPSLE. There are still several questions that need to be answered about the pathogenesis of NPSLE, and better understanding will also lead to the development of improved diagnostic accuracy and the development of therapies that may have better outcomes for our patients.
